# Influence of Environmental Factors and Relationships between Vanadium, Chromium, and Calcium in Human Bone

**DOI:** 10.1155/2016/8340425

**Published:** 2016-05-18

**Authors:** Natalia Lanocha-Arendarczyk, Danuta I. Kosik-Bogacka, Elzbieta Kalisinska, Sebastian Sokolowski, Lukasz Kolodziej, Halina Budis, Krzysztof Safranow, Karolina Kot, Zaneta Ciosek, Natalia Tomska, Katarzyna Galant

**Affiliations:** ^1^Department of Biology and Medical Parasitology, Pomeranian Medical University, Powstancow Wielkopolskich 72, 70-111 Szczecin, Poland; ^2^Chair and Clinic of Orthopaedics, Traumatology and Oncology, Pomeranian Medical University, Unii Lubelskiej 1, 71-252 Szczecin, Poland; ^3^Department of Health Education, University of Szczecin, Piastow 40B, 71-065 Szczecin, Poland; ^4^Department of Biochemistry and Medical Chemistry, Pomeranian Medical University, Powstancow Wielkopolskich 72, 70-111 Szczecin, Poland; ^5^Independent Laboratory of Medical Rehabilitation, Pomeranian Medical University, Żołnierska 54, 71-210 Szczecin, Poland; ^6^Chair of Microbiology and Immunological Diagnostic, Pomeranian Medical University, Powstancow Wielkopolskich 72, 70-111 Szczecin, Poland

## Abstract

The aim of this study was to investigate the impact of environmental factors on the concentrations of vanadium (V), chromium (Cr), and calcium (Ca) and to examine the synergistic or antagonistic relationships between these metals, in cartilage (C), cortical bone (CB), and spongy bone (SB) samples obtained following hip joint surgery on patients with osteoarthritis in NW Poland. We found significantly higher concentrations of V and Cr in spongy bone in patients who consumed game meat and also those with prosthetic implants. Chromium levels were significantly lower in patients with kidney diseases. The greatest positive correlations were found between spongy bone V and (i) the amount of consumed beer and (ii) seafood diet. Correlation analysis also showed a significant correlation between Cr levels and seafood diet. To a certain extent these results indicate that the concentrations of V, Cr, and Ca in the human hip joint tissues are connected with occupational exposure, kidney diseases, diet containing game meat, sea food, beer, and the presence of implants. Furthermore, we noted new types of interactions in specific parts of the femoral head. Vanadium may contribute to the lower bone Ca levels, especially in the external parts (cartilage and cortical bone).

## 1. Introduction

The well-studied long-term accumulation of trace elements as a result of environmental exposure [1–4] can be adequately assessed using bone tissues, thanks to the long-term nature of bone remodeling processes [5]. This information about metal levels in hard tissues can be useful for evaluating nutrition rules and for prevention and control strategies of various diseases caused by imbalances in trace elements [3, 5]. However, analyses of chemical elements in human bones rarely take into account the structural division of bones into cartilage, cortical bone, and spongy bone [5, 6]. This approach neglects the fact that metabolism in the spongy bone is eight times faster than in the cortical bone, resulting in an earlier emergence of pathophysiological changes and faster response to therapy. The literature data also indicate that spongy bone is characterized by higher concentrations of metals compared to other elements of the bone [1, 3, 7, 8].

Vanadium is necessary for metabolic processes including transformations of lipids, phospholipids, and cholesterol [9–12]. The derivatives of V have an antidiabetic effect and act on tumor cells, while supplementation with V stimulates osteogenic cell proliferation and collagen production and increases bone mineral density, mineralization, and formation [13, 14]. On the other hand, excess V may cause biochemical and hematological changes resulting in neurological damage and function impairment of the bones, kidneys, liver, and spleen [15]. Environmental exposure to V compounds is mainly associated with the production of steel, paints, glass, and ceramics. In humans, approximately 50% of V accumulates in the bones, with the remaining part in the kidneys, spleen, liver, blood, adipose tissue, and brain [16]. In 2013, estimated worldwide production of V increased by 5% to 78,200 tons compared with 74,500 tons in 2012 [15, 16]. However, the toxicity of V compounds is low; the lowest is when being consumed with food and the highest when given parenterally. Depending on the study area, the daily intake of V varies from 10 *µ*g to 2 mg. The main sources of V in diet are spices, seafood, fish, beer, and supplements [16].

Chromium (III) is a trace element which stimulates the pancreas and insulin action, facilitates the diffusion of glucose into cells, and is also involved in the metabolism of carbohydrates and proteins [17]. It accumulates in the kidney, liver, and muscle. Chromium deficiency is observed mainly in elderly patients with hypoglycemia or impaired glucose tolerance and type I and II diabetics and also in people on a diet and actively engaged in sport [18]. By contrast, Cr (VI) used in industry has carcinogenic properties. Importantly, the role of Cr in bone metabolism is not yet fully understood [2]. Chromium is widely used in industry as plating, alloying, tanning of animal hides, inhibition of water corrosion, textile dyes and mordants, pigments, ceramic glazes, refractory bricks, and pressure-treated wood [15]. This wide use of Cr has resulted in considerable environmental contamination and has become an increasing concern over the last years [2]. Intoxication of Cr from environmental exposure is not common, except for occupational exposure, but it is suspected that dental implants (prostheses) made of Cr-containing alloys may release chromium into surrounding tissues [2, 15].

Calcium (Ca) is a mineral component which influences the excitability of the nervous system and skeletal and smooth muscles, blood coagulation, and the permeability of cell membranes. The total content of Ca in the human body is from 1.4 to 1.66% of body weight, of which 99% is stored in the bones in the form of hydroxyapatite. Deficient dietary consumption of calcium leads to lower bone mineral content and bone mineral density, and long-term deficiency can lead to rickets, osteomalacia, and osteoporosis [14].

Some researchers suggest that interactions between the various toxic metals and essential macroelements may influence metabolism through exclusion of those metals from biochemical processes. For example, bone calcium has been observed to interact with lead (Pb), nickel (Ni), and copper (Cu) [1, 7]. However, there is no data on the interaction of Ca with elements often present in dietary supplements for the elderly, including V and Cr.

Therefore, the aim of this study was to investigate the impact of environmental factors on the concentrations of V, Cr, and Ca, to examine the synergistic and/or antagonistic relationships between these metals in cartilage, cortical, and spongy bone samples obtained following hip joint surgery on patients with osteoarthritis in NW Poland, and to examine the effects of specific environmental factors: smoking, diet (consumption of fish, seafood diet, dairy products, wild game meat, and alcohol), supplements, including multivitamin/mineral supplements, occupational exposure, and implants.

## 2. Material and Methods

### 2.1. Ethics Statement

This study conforms to the principles outlined in The Declaration of Helsinki as revised in 2008. The use of femoral heads in the investigations was permitted by the Bioethics Committee of the Pomeranian Medical University in Szczecin (BN/001/111/08) and all patients provided written informed consent prior to participation.

### 2.2. Patients

The study material included hip joint sections obtained from patients (*n* = 37) inhabiting urban areas of Western Pomerania. The sections were collected in the Clinic of Orthopaedics and Traumatology at the Pomeranian Medical University in Szczecin as a result of total hip replacement procedures (THR) recommended for HOA-related degenerative changes of the hip not including osteoporosis. In most cases the indication for this treatment was degeneration of the left and/or right hip joint or fracture of the femur (Table 1). Eight of the patients already had cemented or noncemented hip joint prostheses before the hip joint replacement. The cemented prostheses contained nickel-chromium alloys, and the noncemented contained titanium-aluminum-vanadium alloys. All the patients were interviewed using a questionnaire to collect data on demographics, health status, occupational exposure to potentially toxic elements, smoking, and diet (Table 1). Frequency of dietary intake (fish and seafood diet, game meat, dairy products, drinking alcohol (beer), and cigarette smoking) was classified into four levels: none (score 1); sometimes/once a month (score 2); usually/several times a month (3); always/once a day (4). A nutritionist was consulted to create this scale. Although Western Pomerania has no major industrial concentrations, 30–40 years ago (at times when pollution was not well controlled) the patients were occupationally exposed to various contaminants (including contact with chemicals in the shipyards and other workplaces: heavy metals, fertilizers, paints, and reagents used in metallurgy).

### 2.3. Preparation of Bone Tissue Material for Analysis

From each section, three types of material were obtained from the femoral head, cartilage (C), cortical bone (CB), and spongy bone (SB), and were stored at −20°C until analysis. The sampled bones were cleaned of adhering soft tissue and marrow and degreased in acetone (Chempur, Poland) for 3 h. Bone samples were dried to a constant weight at 105°C so that water content could be determined (gravimetric method) and then crushed in an agate mortar. The bone material was mineralized using wet digestion in a Velp Scientifica Mineralizer (Italy). The concentrations of V, Cr, and Ca in the femoral bone head were determined by ICP-AES (inductively coupled argon plasma-atomic absorption spectrophotometry) using a Perkin-Elmer Optima 2000 DV (USA). Its limits of detections (LOD) for V, Cr, and Ca were 0.5, 0.2, and 0.05 g/L, respectively. The concentrations of elements were expressed as mg/kg dry mass (dm) for V and Cr and g/kg dm for Ca.

### 2.4. Validation of Analytical Proceedings

The accuracy of the analytical procedure was monitored by determination of the studied elements in two types of reference materials: NIST SRM 1486 Bone Meal (National Institute of Standards and Technology) and IAEA-407 Trace Elements and Methylmercury in Fish Tissue (International Atomic Energy Agency). The concentration values of the reference materials given by the manufacturers and our determinations are shown in Table 2. In order to determine the possible loss of analyte during the chemical process or the impact of other factors on the results of research, we conducted recovery testing.

### 2.5. Statistical Analysis

Statistical analysis was carried using Statistica PL software. The distribution normality was examined using Shapiro-Wilk tests. To compare the impact of various environmental factors on the concentrations of the analyzed metals in the bone we used a Kruskal-Wallis test, and, in the case of significant differences, a Mann-Whitney test was performed. In addition, we determined the Spearman rank correlation coefficients (*r*_*s*_) between the individual metals in three different parts of the hip joint (C, CB, and SB). In order to determine a possible connection between the studied metals and the environmental factors (cigarette smoking, fish and sea food diet, and alcohol drinking), we calculated *r*_*s*_ and determined its significance. The significance level was *p* < 0.05.

## 3. Results

In cartilage, cortical, and spongy bones, the concentrations of the analyzed elements could be arranged in the following ascending series: V < Cr < Ca (Table 3). With respect to the gender of patients, the concentrations of V were statistically significantly different, approximately 20% lower in cortical bone obtained from the women than from men.

There were no significant differences in the concentrations of metals in the femur head between smokers and nonsmokers. Maximum V value was reported in the cartilage of smokers at 1.39 mg/kg dm. The highest average Cr concentration was observed in the spongy bone of those smoking 10–20 cigarettes per day (0.87 mg/kg dm) and the smallest in the cortical bone of those smoking less than 10 cigarettes per day (0.34 mg/kg dm).

There were no significant differences in the concentrations of metals in the femur head between those who consumed fish and seafood (FD; *n* = 32) and those who did not (NFD). However, the NFD group had only 5 people, which could have strongly affected the outcome of the comparisons. Chromium concentrations in cortical and spongy bone in the FD patients were higher than in the NFD patients, at 0.69 versus 0.47 and 0.87 versus 0.63 mg/kg dm, respectively. The maximum concentration of Ca was recorded in the spongy bone of an FD patient (435.92 g/kg dm). In FD patients, cartilage V concentration was about 1.6 times higher than in the NFD group: 0.43 and 0.27 mg/kg dm. A comparison of the mean levels of chemical elements showed no statistically significant differences between the participants who preferred dairy products and those who did not consume them.

There were significant differences in the V and Cr concentrations in spongy bone between the patients who consumed game meat (GM) and those who did not (NGM) (Table 4). The highest and lowest V and Cr contents in NGM and GM groups were in the spongy bone. The concentration of Ca in the population of GM eaters was the highest in the cartilage and spongy bone and differed significantly (Table 4).

Between the patients occupationally exposed (OE; *n* = 8) or unexposed (UE; *n* = 29) to potentially toxic elements statistically significant differences were observed in various bone tissue sample levels. The contents of V (0.62 mg/kg dm) and Cr (0.85 mg/kg dm) in the OE group in spongy bone were higher than in the UE group (0.43 and 0.61 mg/kg, resp.). There was significant difference in Ca concentration in cartilage, with the lowest in the OE group (162.86 g/kg dm) and the highest in the UE group (227.29 g/kg dm), ~80% more Ca level in cartilage than the OE group.

The lowest Ca concentration in cartilage was detected in the OE group (162.86 g/kg dm) and the highest in the UE group (227.29 g/kg dm) which had ~80% more Ca level in cartilage than the OE group.

There were significant differences in the Cr concentration in spongy bone between the patients with implants (IP; *n* = 8) and without implants (NIP; *n* = 29) (*U* = 59.0, *p* < 0.05), at 0.60 and 0.87 mg/kg dm, respectively. We also observed significant differences in V concentrations in cartilage and spongy bone between the IP and NIP groups (*U* = 48.0, *p* < 0.03 and *U* = 52.0, *p* < 0.05, resp.). The concentrations of V in cartilage and spongy bones in the IP group were higher than in the NIP group at 0.38 versus 0.57 and 0.46 versus 0.64 mg/kg dm, respectively.

There were no statistically significant differences between the patients who used dietary supplements (WS; *n* = 13) and those who did not (NS; *n* = 24). We observed a difference in the V concentration in cortical bone, which was close to statistical significance (*U* = 81.0, *p* = 0.06).

Chromium level was significantly lower in patients who were diagnosed with kidney diseases (KD; *n* = 5). In the group without kidney diseases (WKD; *n* = 32) Cr concentration was 2 times higher than in the KD group, 0.46 and 0.28 mg/kg dm, respectively. The concentration of Cr in spongy bone in the WKD group ranged from 0.17 to 2.36 mg/kg dm, and in those with KD it was in the range 0.34–0.5 mg/kg dm (*p* < 0.01).

The greatest positive correlations were found between the V concentrations and the amount of consumed beer (*r*_*s*_ = 0.83; *p* < 0.001) and with a fish/seafood diet (*r*_*s*_ = 0.72; *p* < 0.001). Correlation analysis showed a significant correlation between Cr concentration and a fish/seafood diet (*r*_*s*_ = 0.69; *p* < 0.04).

Significant positive correlation coefficients were found for the relationships V_C_-V_CB_; V_C_-V_SB_; V_C_-Cr_C_; V_C_-Cr_SB_; V_CB_-V_SB_; V_CB_-Cr_C_; V_CB_-Cr_CB_; V_CB_-Cr_SB_; V_SB_-Cr_CB_; Cr_CB_-Cr_SB_; Cr_CB_-Ca_C_; and Ca_C_-Ca_CB_. Significant negative correlations were found for the relationships V_C_-Ca_CB_; V_C_-Ca_SB_; V_CB_-Ca_SB_; V_SB_-Ca_C_; V_SB_-Ca_CB_; and Cr_C_-Ca_SB_ (Table 5).

## 4. Discussion

The recent years have seen the publication of papers on Cr, V, and Ca levels in bone tissues femoral head, femoral neck, rib bone, and iliac crest of the inhabitants of Europe, North America, Taiwan, China, and the central and nonindustrialized part of Russia (Table 6). Chromium concentrations in human bone ranged from 0.25 to 22.01 mg/kg dm (Table 6), significantly depending on the geographical location; Brodziak-Dopierała et al. [2] observed that Cr contents in the hip joint from patients living in the Upper Silesian Industrial District (USD) were as follows: spongy bones > cartilage > cortical bone, 17.86, 5.73, and 5.33 mg/kg dm, respectively. In this study we observed a similar regularity in Cr levels: cartilage < cortical bone < spongy bone, but the values were an order of magnitude smaller, at 0.45, 0.54, and 0.65 mg/kg dm, respectively. Brodziak-Dopierała et al. [2] investigated the residents of the most industrialized region in Poland where Cr emission in 2005 amounted to 22.3 tons, with the highest level in Poland. The study area in our research, that is, West Pomerania, is an agricultural and industrial area, where the emissions of pollutants into the air from point sources (industrial and energy plants) have tended to decline. Similar levels to those observed in the population from the NW Poland had been observed by Dąbrowski [19] in patients from Poznan (Poland) and Darrah [20] in the US. The concentrations of Cr in the cortical and spongy bone samples from patients living in Poznan were 0.49 and 0.83 mg/kg dm, respectively. In spongy bone samples from US, Cr was present at 0.625 mg/kg dm.

The concentrations of Ca in this study could be arranged in the following ascending series: spongy bone > cortical bone > cartilage. Similar to our research, Brodziak-Dopierała et al. [7, 21] observed that cartilage Ca levels were lower than in the cortical and spongy bones. S. Zaichick and V. Zaichick [6], in the population of the central and nonindustrialized part of Russia, observed lower Ca concentration of iliac crest in women (176 g/kg dm) and men (164 g/kg dm) than in cortical bone in this study: 238.17 g/kg dm and 227.48 g/kg dm, respectively. Little is known about V levels in human hip joint tissues. Studies on the inhabitants of Western Europe and Turkey show high diversity of results (from ~0.05 to 22 mg/kg dm); the levels were determined in the entire femur head (without a structural division) or in rib bone (Table 6). We observed that the highest and the lowest V concentrations were in cortical and cartilage tissues, respectively (Table 3). It is possible that the cartilaginous tissue has a lower storage capacity of this element than the bone tissue. This may indicate that V has accumulative abilities in cortical bone of femoral head.

Diseases such as osteoarthritis (OA), coxarthrosis, osteoporosis, and bone cancers including osteogenic sarcoma may be accompanied by normal, reduced, or elevated Cr, V, and Ca concentrations [6, 21, 22]. Karaaslan et al. [23] in Turkey found that V concentration in patients with OA did not exceed 22 *µ*g/g dm but was two orders of magnitude greater than in patients with OA in this study. Noor et al. [24] showed that V concentration was higher but Cr and Ca were lower in OA patients than without OA subjects. Kuo et al. [18] showed that the average Cr concentration in degenerated femoral bone was 4.96 mg/kg dm, and in ischemic necrosis of the femoral head and osteoarthritis Cr levels were 6.77 and 7.87 mg/kg dm, respectively. Brodziak-Dopierała et al. [2] in the cartilage of patients with OA noted a much higher Cr concentration (4.65 mg/kg dm) than in the OA patients in this study.

Cement prostheses are commonly produced using stainless steel which includes Cr and a small amount of Ni, while the production of cementless prostheses commonly involves titanium-aluminum-vanadium alloys. It has been shown that a damaged prosthesis may release certain ions, including Cr, especially when the overcoat layer of chromium oxide is damaged [15]. This has been confirmed by our observations and differences in Cr and V levels in cartilage and spongy bone between the patients with (IP) and without implants (NIP). However, it is also possible that higher V values resulted from contamination during sampling or sample preparation, when instruments made of stainless steel were used.

Bioaccumulation of chemical elements in the osseous system may be considered in connection with several factors: place of residence, type of the bone disease, cigarette smoking, and dietary preferences.

Cigarette smoking interferes with the carefully controlled metal homeostasis of the human body. The chromium levels in main stream cigarette smoke range from 0.0002 to 0.5 mg per cigarette, although in this study the differences in the concentration of this element were not significant between smokers and nonsmokers. The concentration of V in cigarettes ranged from 0.49 to 5.33 mg/g (average: 1.11 mg/cigarette), yet diet is the major source of exposure to V for the general population [25]. In this study, maximum V level was found in the cartilage of smokers (1.39 mg/kg dm).

Chromium levels in human tissues may be influenced by chromium-rich food that includes lobsters, molluscs, and, to a lesser extent, fish. The main sources of Ca in diet are milk and milk products, while fish contain only low Ca levels, although at high bioavailability. Kuo et al. [18], in a study on a Taiwanese population, observed that bone Cr and Ca concentrations depended on the consumption of seafood/fish, with *r*_*s*_ = 0.31 and *r*_*s*_ = 0.32, respectively. In this study we observed a similar relationship for Cr. However, Cr concentration in human bone in Taiwan was many times higher, because in Taiwan seafood plays an important role in the daily diet, and in Poland dietary habits are also changing in favor of diets richer in seafood and fish. In this study, we observed the maximum Ca concentration > 430 g/kg dm, in the spongy bone of an FD patient. In Poland, the most often consumed fish species are herring, pike, carp, and cod, which contain 32, 20, 10, and 9 mg Ca/100 g, respectively. Łuczyńska et al. [26] measured more than 2 and 3.5 times more Ca in the muscles of carp and trout from Polish fisheries than in Ca derived from salmon muscle [26]. Carp and trout are very popular in Poland, especially during the Christmas season. In this study V concentration in cartilage differed significantly between the FD and NFD groups; however, V levels in fish (especially fish fat) and seafood are one of the highest in any food products.

The consumption of game meat in Poland is low, but this type of diet is dominantly consumed by hunters and their families. We found that, in spongy bone in patients who consumed game meat, V and Cr concentrations reached 0.6 and 0.8 mg/kg dm, respectively, and were significantly higher in people who did not prefer this diet. The content of V in human bones is considered to be the indicator of meat consumption [27]. However, little is known about the V levels in the wild animals tissues; in muscle and organ meats of red deer from Poland the median V concentrations did not exceed 0.016 mg/kg dm [28]. The concentrations of Cr in meat products do not exceed 40 mg/kg wet weight, and most of the Cr in the diet is brought by vegetables (23%) and cereals (20%) and often with pharmaceutical products [29]. In this study, cartilage and cortical bone samples from the GM group had significantly higher Ca levels, as the meat of game animals, especially roe deer, has high levels of magnesium, phosphorus, and (especially) calcium [30]. In Poland Ca levels in organ meats of wild boar, roe deer, and hare are 89.5, 85, and 105.7 *µ*g/g, respectively [31].

A comparison of the mean levels of chemical elements showed no statistically significant differences between the participants who preferred dairy products and those who did not consume them, similar to Kuo et al. [18] in a study in Taiwan. In persons above 65 years of age, the adequate daily dose of Ca is between 1000 and 1500 mg/Ca. The typical Ca absorption from milk is about 30% compared to 5% from spinach and decreases with age. Ca from milk may be supplemented by full grain cereal products and vegetables, but their Ca content is lower; its absorption is also lower due to the presence of caffeine, phytic acid (wheat bran), oxalic acid (spinach, rhubarb), and high phosphorous content [32]. Garcia et al. [33] observed that alcohol-drinking patients living in the region of Tarragona in Spain had a higher Cr concentration; in this study we found that V concentration in the femur head correlated with the consumption of beer (*r*_*s*_ = 0.83). V levels in alcoholic beverages are high; they range from 0.001 to 0.005 mg/100 g in beer, compared to 0.000001–0.0001 mg/100 g in wine. This is significant in the light of the growing consumption of beer and other low-alcohol drinks in Poland [34].

For environmental exposure of the patients from Western Pomerania, the V and Cr contents in the OE group in spongy bone were higher than in the UE group. Statistically significant differences existed in the case of Ca measured in cartilage. The lowest Ca concentration in cartilage was detected in the OE group and the highest in the UE group, but OE group had only 8 people, which could have strongly affected the outcome of the comparisons. Chromium content in bones from the inhabitants of the highly industrialized Taiwan was 11.9 mg/kg dm [18]. It was higher than the average value of all elements of the hip joint in the study by Brodziak-Dopierała et al. [2]. Biological monitoring on the bones of the general population was carried out for patients who had lived at least 10 years in the neighborhood of a hazardous waste incinerator (HWI) in Tarragona (Catalonia, Spain). Vanadium concentration was below the corresponding ~0.05 mg/kg dm, while the Cr concentrations in bone significantly increased [35]. In this research the spongy bone V concentrations in the OE group (0.62 mg/kg dm) were higher than in the UE group (0.43 mg/kg dm) and almost 12 times greater than in a study by Mari et al. [35] in patients exposed to emissions of the Spanish HWI. However Bocio et al. [36] suggested that the emissions of metals by the plant near HWI do not mean additional exposure to these elements for this population. ThesedimentsintheBayofSzczecin(NW Poland) arehighly polluted with V and other elements [37].Dueto riverrunoff,V pollutionoftheBayofSzczecinis comparable tothepollutionofthePersianGulf oilfields [16, 38, 39].

Nielsen [40] reports that V has become a component in a large number of pills and other dietary supplements to enhance strength and ward off diabetes. A number of studies have suggested the ability of vanadium to improve the blood glucose control in diabetics and also to improve the negative side effects associated with diabetes. We observed a difference between the patients who used dietary supplements (WS) and those who did not (NS) in the V concentration in cortical bone, which was close to statistical significance.

Long-term occupational exposure, for example, to Cr (VI), may result in chronic renal failure and the site of the early changes is proximal nephron tubules [41]. D'Haese et al. [42] in the bones of end stage renal failure patients determined that Cr content in the bones was higher in people after dialysis than in people with normal renal function: 0.5 versus 0.2 mg/kg. In this study we also observed a significantly lower Cr concentration in the bones of patients diagnosed with renal disease (KD).

The biochemical roles of Ca and Cr are well known but their relationship with V is less well understood. Most interactions between Cr and Ca were found in the spongy bone, which is associated not only with the physiological function of metals in hydroxyapatite but with the specific mineral structure of the tissue [7, 43]. Brodziak-Dopierała et al. [43] observed a directly proportional correlation between Cr and Ca in spongy bone and cartilage, 0.52 and 0.66, respectively. Moreover, Brodziak-Dopierała et al. [7] demonstrated the interaction between Cr and Ca. In this study we found a significant negative correlation for the relationship Cr_C_-Ca_SB_. This research is also the first to find significant positive correlation coefficients for the relationships V_C_-V_CB_; V_C_-V_SB_; V_C_-Cr_C_; V_C_-Cr_SB_; V_CB_-V_SB_; V_CB_-Cr_C_; V_CB_-Cr_CB_; V_CB_-Cr_SB_; and V_SB_-Cr_CB_. Antagonistic interactions were found between V and Ca for the C/CB, CB/SB, and SB/C (Table 5). This means that probably vanadium may contribute to lower bone Ca levels, especially in its external parts (cartilage and cortical bone).

## 5. Conclusions

The interpretation of our results is difficult and ambiguous as the increased chemical elements concentrations in the bone may have been induced not only by diseases metabolic levels, but also by different environmental factors.

1. Analysis of the impact of environmental factors on V, Cr, and Ca concentrations in the hip joint tissues (cartilage, cortical, and spongy bones) of patients showed the influence of the following:
  - A diet rich in game meat: the highest V and Cr concentrations were found in patients who did not consume game meat. The Ca concentrations in the population of game meat eaters were the highest in the cartilage and spongy bone.
  - Occupational exposure to potentially toxic elements: the highest V and Cr contents were found in patients occupationally exposed (OE), and the lowest Ca concentration was found in the OE group.
  - Prosthetic implants: Cr concentration was the greatest in people without implants, while V concentration was the highest in the patients with implants.
  - Kidney diseases: Cr concentration was lower in patients who were diagnosed with kidney diseases.
2. New types of interactions in specific parts of the femoral head were noted. It was found that V may contribute to lower bone Ca levels, especially in its external parts (cartilage and cortical bone).

## Figures and Tables

**Table 1:** Information on patients included in the study.

Factors	Number of patients (%)	
Biological factors		

Age	Woman	*n* = 24 (64.86) 58–84 (69.90 ± 10.77) years
	Man	*n* = 13 (35.14) 52–79 (62.6 ± 15.4) years

Type of OA	Degeneration of the left hip joint	*n* = 15 (40.54)
	Degeneration of the right hip joint	*n* = 19 (51.35)
	Fracture of the left femur	*n* = 3 (8.11)

Kidney diseases	Patients with diagnosed kidney diseases (KD)	*n* = 5 (13.51)
	Without diagnosed kidney diseases (WKD)	*n* = 32 (86.49)

Environmental factors		

Type of prostheses before second replacement surgery	Cemented hip joint prostheses Noncemented hip joint prostheses Patients without implants	*n* = 6 (16.22) *n* = 2 (5.40) *n* = 29 (78.38)

Cigarette smoking	Smokers Nonsmokers	*n* = 20 (54.05) *n* = 17 (45.95)

Fish and seafood diet	Participant who consumed fish and fish diet (FD)	*n* = 32 (86.49)
	Without this type of diet (NFD)	*n* = 5 (13.51)

Game meat	Participant who consumed game meat (GM)	*n* = 8 (21.62)
	Without this type of diet (NGM)	*n* = 29 (78.38)

Dairy products	Participant who preferred dairy products (DP)	*n* = 32 (86.49)
	Without this type of diet (NDP)	*n* = 5 (13.51)

Alcohol drinking (beer)	Nondrinker Drinker	*n* = 12 (32.43) *n* = 25 (67.57)

Occupational exposure	Occupationally exposed	*n* = 8 (21.62)
	Unexposed	*n* = 29 (78.38)

Dietary supplements	Patients with supplementation (WS)	*n* = 13 (35.14)
	Without supplementation (NS)	*n* = 24 (64.86)

**Table 2:** Table analysis of NIST-SRM 1486 (Bone Meal) and Fish Tissue IAEA-407 by ICP-AES.

Chemical elements	Bone Meal SRM NIST 1486		Recovery (%)	Fish Tissue IAEA-407 (mg/kg dm)		Recovery (%)
	Certified	Measured *n* = 9		Certified	Measured *n* = 10	
Cr	—	—	—	0.73 ± 0.22 (0.67–0.79)	0.56 ± 0.26	76%
V	—	—	—	1.49 ± 0.20 (1.34–1.52)	2.93 ± 0.16	104%
Ca	26.58 ± 0.24 wt. percent	25.69 ± 13.27 wt. percent	96%	27.0 ± 1.8 (25.7–28.3)	17.98 ± 7.25	67%

**Table 3:** The concentrations of V, Cr, and Ca in the cartilage, cortical bone, and spongy bone in women and men with osteoarthritis (AM, arithmetic mean; SD, standard deviation; Med, median, CV, coefficient of variation in percent; *U*, Mann-Whitney *U* test; *p*, level of significance; and NS, nonsignificant difference; V and Cr concentrations are expressed as mg/kg dm; Ca level is expressed as g/kg dm).

Parameter	V	Cr	Ca
Female (*n* = 24)			
Cartilage			
AM ± SD	0.37 ± 0.18	0.40 ± 0.21	221.46 ± 89.74
Med	0.30	0.39	225.69
Range	0.23–0.80	0.16–1.13	57.20–38.78
CV	48.65	52.50	40.52
Cortical bone			
AM ± SD	0.49 ± 0.21	0.59 ± 0.38	238.17 ± 94.90
Med	0.48	0.51	225.52
Range	0.19–0.88	0.18–1.72	110.68–469.20
CV	42.86	64.41	39.84
Spongy bone			
AM ± SD	0.51 ± 0.30	0.64 ± 0.54	245.79 ± 96.36
Med	0.35	0.43	242.04
Range	0.23–1.28	0.18–2.36	60.18–435.92
CV	58.82	84.37	39.20

Male (*n* = 13)			
Cartilage			
AM ± SD	0.50 ± 0.37	0.52 ± 0.48	200.87 ± 73.38
Med	0.32	0.34	213.15
Range	0.20–1.40	0.14–1.57	78.08–311.64
CV	74.0	93.31	36.53
Cortical bone			
AM ± SD	0.64 ± 0.23	0.80 ± 0.47	227.48 ± 98.70
Med	0.60	0.68	223.83
Range	0.31–1.05	0.22–1.91	79.70–417.30
CV	35.94	58.75	43.39
Spongy bone			
AM ± SD	0.48 ± 0.26	0.69 ± 0.70	243.19 ± 84.64
Med	0.34	0.37	249.46
Range	0.23–1.02	0.21–2.25	116.25–386.01
CV	54.17	101.45	34.80

Total (*n* = 37)			
Cartilage			
AM ± SD	0.42 ± 0.26	0.45 ± 0.33	214.40 ± 83.97
Med	0.30	0.36	223.80
Range	0.20–1.40	0.14–1.57	57.20–387.78
CV	61.90	73.33	39.16
Cortical bone			
AM ± SD	0.54 ± 0.23	0.66 ± 0.42	234.28 ± 94.90
Med	0.57	0.54	225.52
Range	0.19–1.05	0.18–1.91	79.70–469.20
CV	42.59	63.63	40.51
Spongy bone			
AM ± SD	0.50 ± 0.30	0.66 ± 0.60	244.92 ± 91.40
Med	0.35	0.41	243.70
Range	0.23–1.28	0.18–2.36	60.18–435.92
CV	15.0	90.91	37.32

Female versus male			
Cartilage			
*U*	NS	NS	NS
*p*			
Cortical bone			
*U*	91	NS	NS
*p*	0.07		
Spongy bone			
*U*	NS	NS	NS
*p*			

**Table 4:** The concentrations of V, Cr, and Ca in the cartilage, cortical bone, and spongy bone in patients consuming game meat (GM) and without this type of diet (NGM) (AM, arithmetic mean; SD, standard deviation; Med, median; CV, coefficient of variation in percent; *U*, Mann-Whitney *U* test; *p*, level of significance; and NS, nonsignificant difference; V and Cr concentrations are expressed as mg/kg dm; Ca level is expressed as g/kg dm).

Parameter	V	Cr	Ca
NGM (*n* = 29)			
Cartilage			
AM ± SD	0.44 ± 0.29	0.46 ± 0.36	199.90 ± 84.82
Med	0.30	0.36	207.22
Range	0.20–1.40	0.14–1.57	57.20–387.78
CV	65.91	78.26	42.43
Cortical bone			
AM ± SD	0.56 ± 0.24	0.72 ± 0.46	215.76 ± 89.60
Med	0.57	0.58	212.00
Range	0.19–1.05	0.18–1.91	79.70–469.20
CV	42.86	63.89	41.53
Spongy bone			
AM ± SD	0.55 ± 0.30	0.75 ± 0.64	249.03 ± 96.34
Med	0.42	0.47	263.99
Range	0.23–1.28	0.18–2.36	60.18–435.92
CV	54.54	85.33	38.69

GM (*n* = 8)			
Cartilage			
AM ± SD	0.33 ± 0.06	0.39 ± 0.17	263.33 ± 63.10
Med	0.30	0.36	261.17
Range	0.28–0.46	0.22–0.77	150.00–369.08
CV	18.18	43.59	23.96
Cortical bone			
AM ± SD	0.46 ± 0.14	0.49 ± 0.19	292.18 ± 92.65
Med	0.45	0.47	267.44
Range	0.31–0.65	0.22–0.77	171.26–439.91
CV	30.43	38.77	31.71
Spongy bone			
AM ± SD	0.31 ± 0.08	0.31 ± 0.08	230.52 ± 75.31
Med	0.32	0.33	228.44
Range	0.23–0.47	0.20–0.42	135.89–386.01
CV	25.81	25.81	32.67

NGM versus GM			
Cartilage			
*U*	NS	NS	51
*p*			0.02
Cortical bone			
*U*	NS	NS	52
*p*			0.04
Spongy bone			
*U*	54	46	NS
*p*	0.03	0.01	

**Table 5:** Spearman rank correlation coefficients between three elements (V, Cr, and Ca) determined in various types of bone material (*n* = 37) of patients with osteoarthritis following hip joint arthroplasty (C, cartilage; CB, compact bone; SB, spongy bone).

	V_C_	V_CB_	V_SB_	Cr_C_	Cr_CB_	Cr_SB_	Ca_C_	Ca_CB_	Ca_SB_
V_C_	—								
V_CB_	0.57^xxxx^	—							
V_SB_	0.49^xxx^	0.54^xxxx^	—						
Cr_C_	0.60^xxxx^	0.40^xx^	NS	—					
Cr_CB_	0.40^xx^	0.71^xxxx^	0.37^xx^	NS	—				
Cr_SB_	0.33^xx^	0.51^xxx^	0.80^xxxx^	NS	0.29^xx^	—			
Ca_C_	NS	NS	−0.42^xxx^	NS	0.50^xx^	NS	—		
Ca_CB_	−0.31^xx^	NS	−0.40^xx^	NS	NS	NS	0.40^xx^	—	
Ca_SB_	−0.40^xx^	−0.32^xx^	NS	−0.55^xxx^	NS	NS	NS	NS	—

^xx^ *p* < 0.05; ^xxx^ *p* < 0.01; ^xxxx^ *p* < 0.001; NS, nonsignificant difference.

**Table 6:** Mean concentrations of Cr, V, and Ca in bone material coming from patients from various countries, based on the literature (HWI, hazardous waste incinerator; OA, osteoarthritis; OSB, osteoporotic bone; RF, renal failure; IB, intact bone; ES, Ewing's sarcoma; USIA, Upper Silesian Industrial Area).

Number	Origin of bone material	Age	Sex	*n*	dm/wm	Cr	V	Ca	Source
	Femur head (with a structural division)								

	Cartilage								

(1)	Poland, Western Pomerania	32–84	F + M	37	mg/kg dm	F 0.40 M 0.52	F 0.37 M 0.50	g/kg F 221.46 M 200.87	This study
(2)	Poland, USIA	63.9	F + M	26	*µ*g/g dm	2.50		21,308.89	[43]
(3)		71	F + M	103	*µ*g/g dm	16.56	—	30,917.40	[1]
(4)		65.6	F + M	53	mg/g dm	—	—	F 27.81 M 25.48	[1]
(5)		65.7	F + M	84	*µ*g/g dm	5.85 OA	—	—	[9]
(6)		65.7	F + M	84	mg/g dm	—	—	23.65 (3.56–96.66)	[21]
(7)		54–86	F + M	37	*µ*g/g dm	F 3.43 M 7.02	—	—	[44]
(8)	China, Shanghai	18–30 55–76	F + M	7/O 5/K	*µ*g/g dm	—	—	32800	[45]

	Cortical bone								

(9)	Poland, Western Pomerania	32–84	F + M	37	mg/kg dm	F 0.49 M 0.64	F 0.59 M 0.80	g/kg F 238.17 M 227.48	This study
(10)	Poland, USIA	63.9	F + M	26	*µ*g/g dm	3.45	—	25,212.61	[43]
(11)		71	F + M	103	*µ*g/g dm	14.99	—	30,216.83	[1]
(12)		65.6	F + M	53	mg/g dm	—	—	F 48.97 M 49.61	[7]
(13)		65.7	F + M	84	*µ*g/g dm	5.07 OA	—	—	[2]
(14)		65.7	F + M	84	mg/g dm	—	—	43.52 (1.24–83.61)	[21]
(15)		54–86	F + M	37	*µ*g/g dm	F 1.81 M 1.33	—	—	[44]
(16)	China, Shanghai	18–30 55–76	F + M	7/O 5/K	*µ*g/g dm	—	—	228,800	[45]

	Cortical bone from the femur and tibia								

(17)	Russia, Obninsk	7–25	F + M	20	g/kg dm	—	—	218 (IB) 207 (O) 81.3 (ES)	[46]
(18)	Taiwan	41–80	F + M	77	mg/kg dm	F 22.01 M 6.48		F 77,115 M 84,649	[18]

	Spongy bone								

(19)	Poland, Western Pomerania	32–84	F + M	37	mg/kg dm	F 0.51 M 0.48	F 0.64 M 0.69	g/kg F 245.79 M 243.19	This study
(20)	Poland, USIA	63.9	F + M	26	*µ*g/g dm	2.89	—	27,929.17	[43]
(21)		65.6	F + M	53	mg/g dm	—	—	F 160.73 M 160.12	[7]
(22)		65.7	F + M	84	*µ*g/g dm	16.38 OA	—	—	[2]
(23)		65.7	F + M	84	mg/g dm	—	—	127.48 (27.17–184.4)	[21]
(24)		54–86	F + M	37	*µ*g/g dm	F 3.06 M 5.43	—	—	[44]
(25)		71	F + M	103	*µ*g/g dm	10.42	—	25,244.65	[1]
(26)	China, Shanghai	18–30 55–76	F + M	7/O 5/K	*µ*g/g dm	—	—	132,300	[45]

	Femur head (without a structural division)								

(27)	Turkey, Erciyes	50–70	F + M	30	*µ*g/g dm	10.0	22.0	13.9 OA	[23]
(28)	Poland, USIA	65.6	F + M	53	*µ*g/g dm	7.58 OA (0.05–89.14)	—	49,485.44 OA (378.09–184,396.05)	[7]
(29)	Taiwan	41–60 61–80	F + M	70	*µ*g/g dm	11.9	—	82,007.9	[18]
(30)	Poland, USIA, Lodz, Cracow	45–79 47–86 50–80	F + M	38	%	—	—	17.44 16.95 20.70	[47]
(31)	Poland, USIA	65.8	F + M	197	%	—	—	17.01 (7.27–34.01)	[48]

	Femoral neck								

(32)	Russia, Obninsk	15–55	F + M	84	g/kg dm	—	—	162	[8]

	Rib bone								

(33)	Spain, Tarragona, HWI	–	F + M	22	*µ*g/g dm	1998 0.51 2003 0.24	1998 <0.12 2003 <0.25		[36]
(34)	Spain, Tarragona, HWI	51	F + M	20	*µ*g/g wm	2007 1.39 2013 1.38	2007 <0.25 2013 <0.25	—	[35]
(35)	Russia, Obninsk	15–55	F + M	38	mg/kg dm	<0.25 (<0.20–1.6)	—	—	[37]
(36)		15–55	F + M	84	g/kg wm	—	—	116 64.6–222	[49]
(37)		15–65	F + M	80	mg/kg dm	—	<0.03	171.395 85.193–233.624	[8]
(38)	Japan, Tokyo	61–96	F + M	45	*µ*g/g dm	<6.0	<0.08	246 mg/g	[22]

	Transiliac bone								

(39)	Belgium, Greece, Czech, Argentina, Egypt	58.8	M	100	*µ*g/g wm	—	—	203.0 RF	[42]

	Iliac crest								

(40)	Russia, Obninsk	15–55	F + M	84	g/kg dm	—	—	162	[6]

	Bone								

(41)	Indonesia, Banjarmasin and Malang	>50	F	32	*µ*g/g dm	1.28 OSB	—	58,150.53	[24]
(42)	Korea, Seoul	13–87	F + M	162	*µ*g/g wm	0.27	0.49		[50]
